# Complexation of Manganese with Glutarimidedioxime: Implication for Extraction Uranium from Seawater

**DOI:** 10.1038/srep43503

**Published:** 2017-03-07

**Authors:** Xiang Xie, Yin Tian, Zhen Qin, Qianhong Yu, Hongyuan Wei, Dongqi Wang, Xingliang Li, Xiaolin Wang

**Affiliations:** 1 Institute of Nuclear Physics and Chemistry, China Academy of Engineering Physics, Mianyang, Sichuan 621999, China; 2 Southwestern Institute of Physics, Chengdu, Sichuan 610041, China; 3 Institute of Materials, China Academy of Engineering Physics, Mianyang 621900, China; 4 ^4^CAS Key Laboratory of Nuclear Radiation and Nuclear Energy Techniques, and Institute of High Energy Physics, Chinese Academy of Sciences, Beijing, 100049, China

## Abstract

The molecule of glutaroimidedioxime, a cyclic imidedioxime moiety that can form during the synthesis of the poly(amidoxime)sorbent and is reputedly responsible for the extraction of uranium from seawater. Complexation of manganese (II) with glutarimidedioxime in aqueous solutions was investigated with potentiometry, calorimetry, ESI-mass spectrometry, electrochemical measurements and quantum chemical calculations. Results show that complexation reactions of manganese with glutarimidedioxime are both enthalpy and entropy driven processes, implying that the sorption of manganese on the glutarimidedioxime-functionalized sorbent would be enhanced at higher temperatures. Complex formation of manganese with glutarimidedioxime can assist redox of Mn(II/III). There are about ~15% of equilibrium manganese complex with the ligand in seawater pH(8.3), indicating that manganese could compete to some degree with uranium for sorption sites.

Nuclear power is an important alternative energy to fossil fuels. One gram of ^235^U can theoretically produce, through nuclear fission, as much energy as burning 1.5 million grams of coal. But uranium resources on land are not unlimited. The total amount of uranium in the ocean is estimated to be 4.5 billion metric tons, more than a thousand times as much as that in terrestrial ores. Developing techniques for extracting uranium from seawater is attracting considerable interest because of the huge demand in uranium as a fuel in the nuclear energy systems. However, extraction of uranium from seawater is extremely challenging. Uranium is present at an extremely low concentration (3.3 μg·L^−1^) in the forms of very stable triscarbonato complex, [UO_2_(CO_3_)_3_]^4−^1. More recent studies suggest that this complex is further stabilized by the formation of ternary complexes with calcium and magnesium due to the overwhelmingly high concentrations of calcium, magnesium, and carbonate in seawater^2^,^3^. Many other cations including transition metal ions also exist in seawater, some of which are in concentrations higher than or comparable to that of uranium^4^. Therefore, the process for the capture of uranium from other more abundant metal ions requires high affinity, selectivity and the ability to deal with an enormous volume of water.

Various techniques have been studied and developed for the extraction of uranium from seawater^5^,^6^,^7^,^8^,^9^,^10^,^11^,^12^,^13^,^14^,^15^. Screening studies conducted in the 1980s with more than 200 functionalized adsorbents showed that sorbent materials with the amidoxime group R-C(NH_2_)(NOH) were most effective for uranium adsorption from seawater. Recent research efforts in Japan, China, and the USA have focused on using amidoxime-based adsorbents for sequestering uranium from seawater. The amidoxime-based fiber can be prepared by a radiation-induced graft polymerization method which involves grafting of acrylonitrile (CH_2_=CH–CN) onto polyethylene fabrics and chemical conversion of the –CN groups with hydroxylamine to the amidoxime groups. This type of sorbents show good mechanical strength and a high capacity for uranium sorption from seawater. The amidoxime groups formed in the polymer sorbent by the synthesis method described above may exist in two different structures as illustrated in Fig. 1. Both the cyclic imide dioxime and the open-chain diamidoxime (Fig. 1) on the sorbent can form strong complexes with uranium. Linfeng *et al*. recently reported that the open-chain diamidoxime is a weaker competing ligand than the cyclic imide dioxime (denoted as H_2_A in this paper) for complexation with UO_2_^2+^ under the seawater conditions^16^,^17^.

Due to the expansion of nuclear power, there is an urgent increasing demand for uranium in China. From a strategic point of view, we should focus on the development of seawater uranium extraction, at least as a technical reserve to ensure steady supply of uranium in future. Amidoxime-based fiber materials have been developed in our institute for the seawater tests in South China Sea. The results from marine test show that the sorption of vanadium, barium, manganese, and chromium ions are comparable or even higher than that of uranium.

Thermodynamic studies of the complexation of simple amidoxime ligand with uranium and other seawater cations can be used to aid the development of sorbents that are more efficient, more selective, and more robust. Because of their importance in providing fundamental information on the nature (e.g., ionic bonding *vs* covalence bonding, outer sphere *vs* inner sphere), energetics (e. g., free energy, enthalpy, entropy and heat capacity), structures and stabilities of complexes, thermodynamic and structural studies have been carried out to understand scientific basis for efficient extraction of uranium from seawater^18^,^19^. Data indicated that the binding strength of glutarimidedioxime with the metal cations in seawater followed the order: Fe^3+^ > UO_2_^2+^ ~ Cu^2+^ > Pb^2+^ > Ni^2+^ > Ca^2+^ ~  Mg^2+^^17^,^20^,^21^. Previous thermodynamics studies do not include Ba^2+^, Mn^2+^, and Cr^3+^, the competing ions for the sequestration of uranium from seawater. To provide thermodynamic data to help evaluate the competition of Mn^2+^ with UO_2_^2+^ in the sequestration process for sorption, stability constants and enthalpies of complexation for the complexes of Mn^2+^ with glutarimiddioxime are needed.

Therefore, the present work has been conducted with a focus on the complexation of Mn^2+^ with glutarimidedioxime by potentiometric, calorimetric, electrochemical experiments and density functional theory (DFT) study. Using the thermodynamic data for Mn^2+^, in conjunction with the data for uranium and other cations from previous studies, the completion of all major cations in seawater with uranium for sorption can be quantitatively evaluated.

## Results and Discussion

### Stability constants

**Figure 2:**
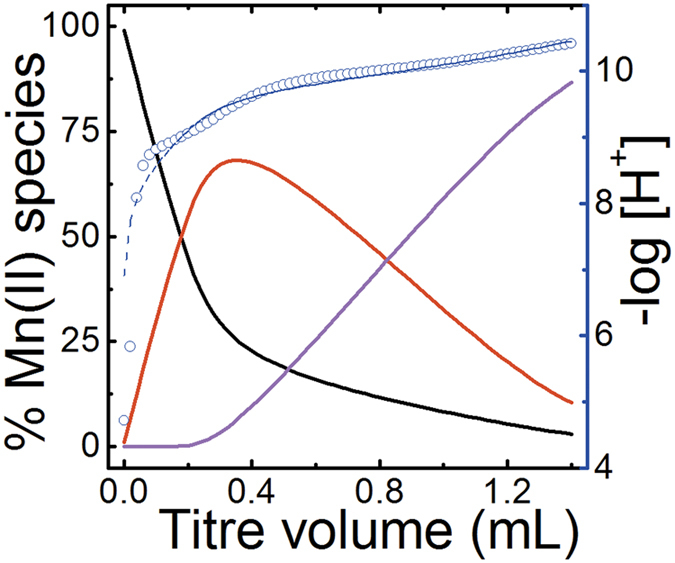
Representative potentiometric titrations for the complexation of H_2_A with Mn(II) at 25 °C(H_2_A stands for the neutral glutarimidedioxime), *I* = 0.5 mol∙L^−1^ NaCl, Initial solution: *V* = 26.0 mL, *n*_Mn_^2+^ = 20.58 μmol, *n*_A_^2−^ = 44.35 μmol, *n*_H_^+^ = 88.48 μmol, Titrant: 63.55 mmol·L^−1^ NaOH, Symbols: blue open circle – experimental data (−log[H^+^]), blue dashed line – fit (−log[H^+^]), solid lines – percentages of species relative to the total Mn(II) concentration (black: free Mn^2+^, red: MnHA^+^, pink: MnA_2_^2−^).

Representative potentiometric titrations at 25 °C and the fitting curves are shown in Fig. 2. The best model to fit the potentiometric data includes the formation two successive Mn(II) complexes, MnHA^+^ and MnA_2_^2−^, as represented by Eqs (1 and 2):


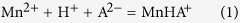



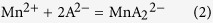


**Figure 3:**
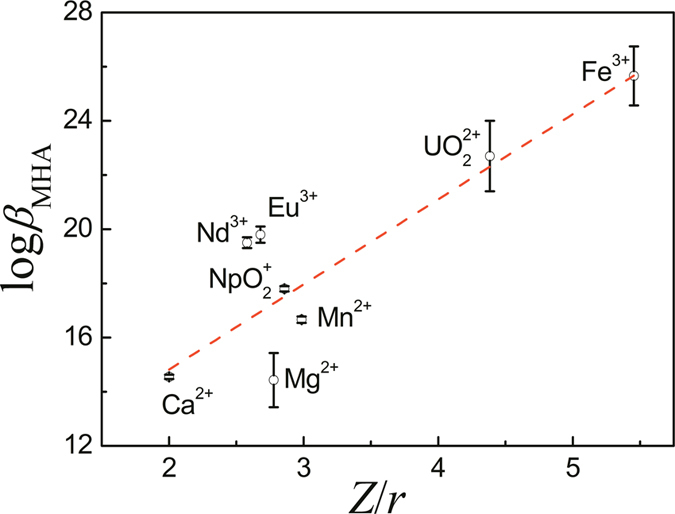
Equilibrium constants of MHA complexes vs ionic potential (*Z*/*r*).

From multiple titrations, the calculated stability constants (log *β*) for MnHA^+^ and MnA_2_^2−^ are listed in Table 1. The results show that the manganese complexes with glutarimidedioxime are much weaker than the uranyl complexes by six to nearly fifteen orders of magnitude. The stability constants of Mn^2+^ complexes with glutarimidedioxime are also lower than those of the transition metal complexes^20^, but much stronger than that of Ca^2+^ or Mg^2+^ complexes with glutarimidedioxime^21^. For all metal cations studied for the complexation with glutarimidedioxime, the stability constants follow the order: Fe^3+^ > UO_2_^2+^ > Eu^3+^~Nd^3+^ > NpO_2_^+^ > Mn^2+^ > Ca^2+^~Mg^2+^. The order is in excellent agreement with the order of ionic potential, *Z*/*r*, where *Z* is the effective charge and *r* is the ionic radius of the ion. A linear correlation between log*β*_MHA_ and *Z*/*r* in Fig. 3 suggests that the interactions between glutarimidedioxime and these ions are predominatly ionic in nature. The stronger complexation of UO_2_^2+^ with the ligand than that of Mn^2+^ could be due to the difference in the effective charges, in addition to the possible overlap of the ligand orbital with the *f*-orbital of uranium. In fact, the effective charge on UO_2_^2+^ is 3.2 as shown in the literature^22^, and much higher than that on Mn^2+^. The high-spin configuration of the Mn^2+^ ion does not provide ligand field stabilization energy and the stability constants of its complexes are consequently lower than those of corresponding complexes of neighboring divalent metal ions^23^.

### Enthalpy of complexation

**Figure 4:**
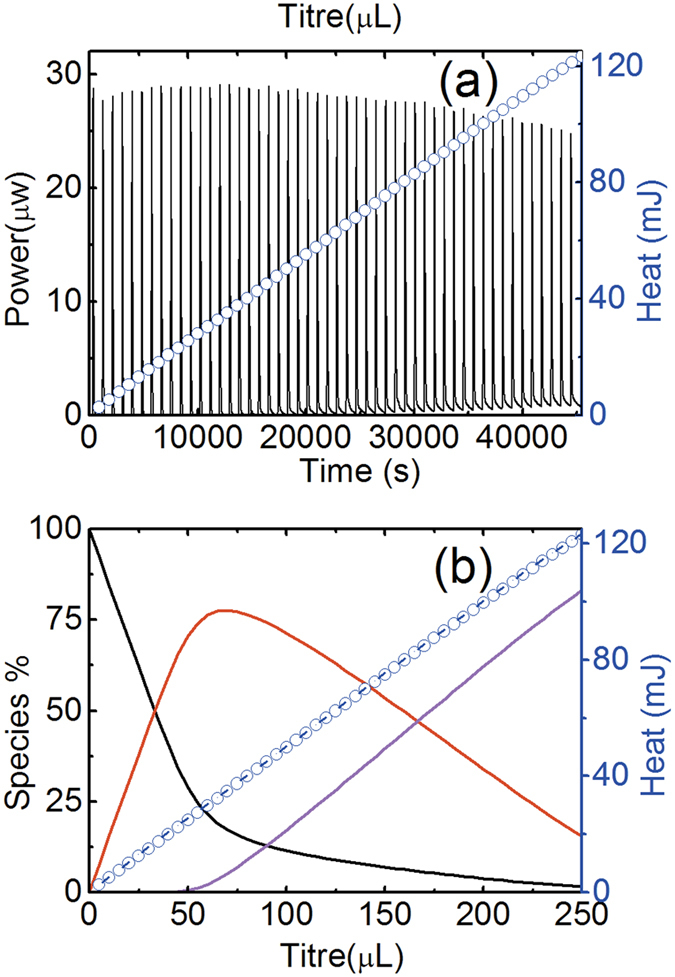
Calorimetric titrations for the complex formation of glutarimidedioxime with Mn(II) at 25 °C. *I* = 0.5 mol∙L^−1^ NaCl. (**a**) Thermogram (solid line, using bottom x-axis and left y-axis) and cumulative heat (blue circule symbols, using top x-axis and right y-axis). (**b**) Cumulative heat (right y axis, blue circule symbols - experimental *Q*, dashed line – fitted *Q*) and speciation of Mn(II) (left y axis, black: free Mn^2+^, red: MnHA^+^, pink: MnA_2_^2−^, where H_2_A stands for the neutral glutarimidedioxime) vs. the titrant volume. Initial cup solutions: *V* = 750 μL, 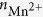 = 1.001 μmol, 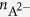 = 3.023 μmol, 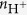 = 6.046 μmol, Titrant: 15.89 mmol·L^−1^ NaOH, injection volume: 5.0 μL.

Data of the calorimetric titrations for the complexes of glutarimidedioxime with Mn(II) are shown in Fig. 4. Using the stoichiometric concentrations of the reactants and the stability constants measured by potentiometry in this work, the enthalpies for the complex formation glutarimidedioxime with Mn(II) at 25 °C are calculated from the calorimetric titration data, and are presented in Table 1. The results show that the enthalpies of MnHA^+^ and MnA_2_^2−^ are both exothermic. The calculated entropies of complexation are all positive. The large positive entropies of complexation probably result from the release of water molecules from the primary coordination spheres of Mn^2+^. In particular, the degree of disorder in the primary hydration sphere is expected to increase significantly if charge neutralization accompanies the formation of complexes.

### Verification of the formation of Mn(II) complexes by ESI-MS

The 1:2 and 1:3 Mn/A complexes were observed in the MS spectrum at *m/z* of 340 and 483 in form of [Mn(A)_n_(A - H)]^+^ (n = 1–2, A stands for the neutral glutarimidedioxime), as the result of the charge reduction reaction (Figure S1). In contrast, 1:1 Mn/A complex could not be directly obtained. The higher abundance of 1:2 Mn/A complex relative to that of 1:3 Mn/A complex suggested the higher stability of the former in the gas phase. This is further rationalized by their fragmentation in tandem MS. In detail, the 1:3 Mn/A complex at *m/z* of 483 dissociated by losing the neutral glutarimideoxime ligand, leaving the 1:2 Mn/A complex as the only fragment ion. With the same collision energy, the 1:2 Mn/A complex fragment into 1: 1 Mn/A complex at *m/z* of 197, as well as the other of the fragment ions due to the fragmentation of the ligand itself such as dehydration reactions so that the binding between the ligands and manganese ion were retained as large as possible. Although the quantitative speciation of the solution by MS is difficulty, these results indicate the consistency between the aqueous phase and the gas phase^24^,^25^.

### Molecular Modeling (DFT Calculation)

**Figure 5:**
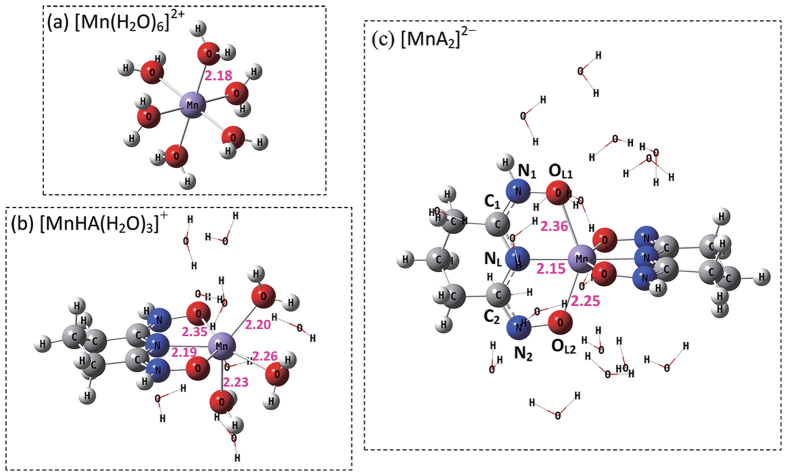
The optimized structures of the stationary points for coordination complex ([MnHA(H_2_O)_3_]^+^ and [MnA_2_]^2−^) by DFT calculations.

Attempts to prepare crystals of the Mn(II)/glutaroimide dioxime complexes were not successful. DFT Calculations were carried out to gain deeper insights into coordination geometry. The crystal structure of V(V), U(VI), Eu(III) and Fe(III) complex with glutarimidedioxime reveals that the ligand bind in a tridentate mode via the imide nitrogen and both oxime oxygens^17^,^20^,^26^,^27^. In these metal complexes, the imide nitrogens are deprotonated while the protons on both oxime oxygens are shifted onto the oxime N atoms. It is reasonable to assume that the ligand also coordinates to Mn^2+^ in a tridentate mode. We hypothesize a reaction mode for complexation of Mn(II) with glutaimidedioxime as Figure S2. Theoretical geometry optimization and the important bond lengths are shown in Fig. 5. In aqueous solution, Mn^2+^ forms a stable hydrated ion that contains six water molecules in the first hydration shell (Fig. 5a)^28^,^29^. The complex formation is an exchange process in essence between the glutarimidedioxime and the coordinated water molecules in the first hydration shell of the Mn(II)^30^. The most practical solvation model should comprise the explicit inclusion of waters in the first hydration shell combined with continuum solvation model for the remainder of the hydration shell^31^. DFT calculations of the thermodynamic parameters for the complexation are summarized in Table S1. The results are generally consistent with the enthalpies directly measured in this work. Structure of the 1:1 and 1:2 metal-ligand complexes, [Mn(HA)(H_2_O)_3_]^+^ and [MnA_2_]^2−^, are also shown in Fig. 5b and c. The bond lengths of glutarimidedioxime ligand before and after coordination obtained by the DFT calculations are summarized in Table S2. When the oxime N-O bond lengths in the metal complex (from 1.354 to 1.391 Å) are compared with those determined from the crystal structure of the bare ligand (H_2_A: 1.42 Å), the oxime N-O bond lengths in metal complexes were noticeably shorter than those in H_2_A and were indicative of conjugation within the ligand in complexes of [Mn(HA)(H_2_O)_3_]^+^ and [MnA_2_]^2−^. The changes of Mayer bond orders (MBOs) (Table S3) suggest that the ligand in [MnA_2_]^2−^ is slightly more conjugated than in [Mn(HA)(H_2_O)_3_]^+^. The natural negative charge of the N donor in [Mn(HA)(H_2_O)_3_]^+^ is higher than that in [MnA_2_]^2−^. The Mulliken charges (Table S3) on the Mn(II) in complexes of [Mn(HA)(H_2_O)_3_]^+^ and [MnA_2_]^2−^ were calculated to be 1.407 and 1.294, respectively, indicating donation of 0.113 e^−^ from the ligand to Mn(II). This indicates that more electron density is donated from the N donor to Mn(II) in [MnA_2_]^2−^ than in [Mn(HA)(H_2_O)_3_]^+^, implying a higher degree of orbital overlapping between the liangd and Mn(II) in [MnA_2_]^2−^. These results reflect a slightly greater covalent character of [MnA_2_]^2−^ than [Mn(HA)(H_2_O)_3_]^+^ complex.

### Electrochemistry

**Figure 6:**
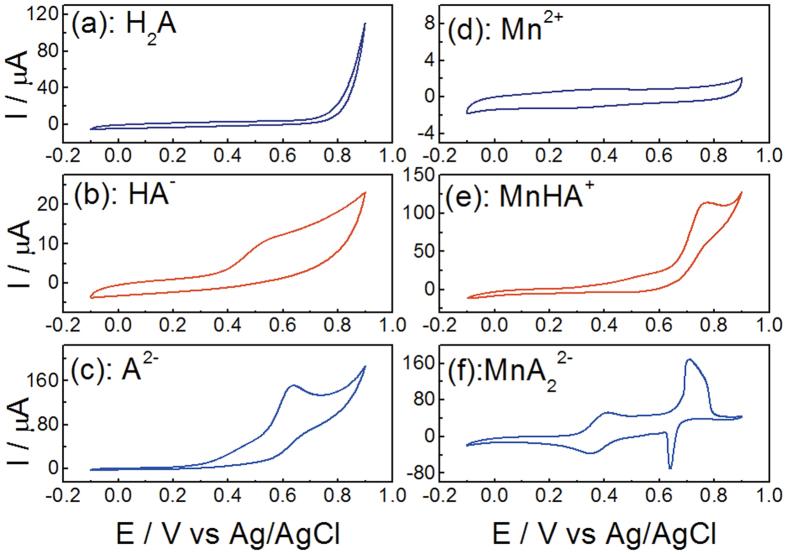
Cyclic voltammograms of 0.5 mol· L^−^ NaCl solution containing H_2_A, HA^−^, A^2−^, Mn^2+^, MnHA^+^, and MnA_2_^2−^. (**a**) pH 4.1, H_2_A to total ligand 99%; (**b**): pH 11.5, HA^−^ to total ligand 70%; (**c**): pH 13.3, A^2−^ to total ligand 95%; (**d**): MnCl_2_ in HCl solution, Mn^2+^ 203.7 mmol·L^−^, H^+^ 218.1 mmol·L^−^; (**e**): pH 8.9, MnHA^+^ to total ligand 81%, MnHA^+^ to total Mn(II) 74%; (**f**): pH 10.1, MnA_2_^2−^ to total ligand 75%, MnA_2_^2−^ to total Mn(II) 97%.

Cyclic voltammetry (CV) measurements were performed to understand the influence that ligand electronic and structural changes exert over redox response. Figure 6 shows typical CV recorded with a glassy carbon working electrode. There is no electrochemical response was observed when the H_2_A solution was scanned between −0.1 and 0.9 V. By comparison of parts in Fig. 6a,b and c, the deprotoned ligand displays a chemically irreversible oxidation wave between 0.5 and 0.64 V. This can be explained by that deprotoned oxime group attribute to more electron-releasing effect. Mn^2+^ in acidic solution is redox innocent within the potential window analyzed (Fig. 6d). The resistance of Mn^2+^ to both oxidation and reduction is generally attributed to the effect of the symmetrical d5 configuration^29^. There is no doubt that the steady increase in resistance of divalent metal ions to oxidation found with increasing atomic number across the first transition series suffers a discontinuity at Mn^2+^, which is more resistant of oxidation than either Cr^2+^ to the left or Fe^2+^ to the right^29^. The wave at 0.77 V in Fig. 6e is similar to that in Fig. 6c, presumably due to oxidation of the ligand in complex of MnHA^+^. The shift of oxime proton onto the oxime nitrogen can be responsible for oxidation wave in Fig. 6e. Mulliken charges on the oxime O and imide N in the free HA^−^ ligand are −1.03 and −0.66, respectively^17^. In comparison, for the ligand HA^−^ in the [Mn(HA)(H_2_O)_3_]^+^ complex, the calculated Mulliken charges on the oxime O and imide N are –0.743 and −0.519 (Table S3), respectively. It can ascribe the more positive potential in Fig. 6e than 6c to the lower charged ligand around the Mn(II) ion and a conjugated system in complex of MnHA^+^ because such a configuration in the (HA)^−^ moiety could allow for a conjugated system in which the electrons are delocalized throughout the -O-N-C-N-C-N-O- bonds. So, the ligand in MnHA^+^ complex is expected to be oxidized at a higher potential. In Fig. 6f, reversible redox wave for [MnA_2_]^2−^ complex between 0.35 and 0.41 V occurring near the midpoint of the quasi-reversible Mn^II/III^ redox couples and thus are attributed to Mn(II/III) activity^32^. It is known that Mn(II/III) redox events are influenced by the electron-releasing properties of coordinated ligand^33^. Free Mn^3+^ generated on the anode surface immediately disproportionate, and the quasi-reversible redox couple of uncomplexed Mn(II/III) redox couple is very hard to be observed. In this work, Mn(III) oxidation state is stabilized significantly by complexed with two ligands and the complexed Mn(II/III) redox couple gives rise to the quasi-reversible redox couple. This observation supports the interpretation of a complex-assisted redox of Mn(II/III). The second waves are mixed with the oxidation of the two deprotoned ligands in [MnA_2_]^2−^ complex and metal-based oxidation process corresponding to Mn(III)/Mn(IV). Most of the manganese complexes isolated at these high valent oxidation states are multinuclear, the dominant forms consisting of oxo, alkoxo, and carboxylato bridged di-, tri-, and tetranuclear. So, the reactivity probably involves reaction with hydroxide ion. Similar reactivity with water has been observed for Mn(III)/Mn(IV) redox couples with highly positive potential^34^.

### Influence of Mn^2+^ complexation with glutarimidedioxime on the extraction of uranium from seawater

**Figure 7:**
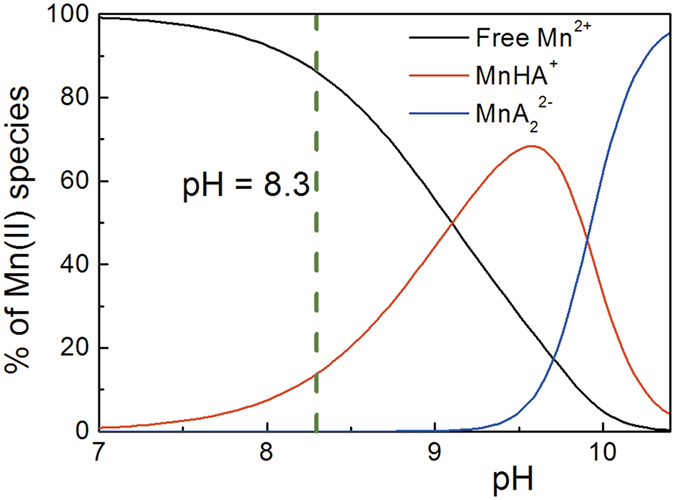
Speciation of Mn(II) as a function of pH (25°C and *I* = 0.5 M). *C*_A_ = 0.001 M, *C*_Mn(II)_ = 4 μg·L^−1^.

Using the thermodynamic data from this work, a speciation diagram (Fig. 7) is drawn for a solution containing manganese at the concentration in seawater (i.e. 4 μg·L^−1^) and 0.001 mol·L^−1^ glutarimidedioxime^1^. The speciation diagram shows that the complex formation of manganese with glutarimidedioxime depends on the pH. There are about ~15% of equilibrium manganese complex with glutarimidedioxime at seawater pH (8.3). It is expected that a significant fraction of the sorption sites could be occupied by manganese as long as there are sufficient sorption sites are available. The results from our institute and other early marine tests for the extraction of uranium have shown that fairly quantities of manganese were sorbed onto amidoxime-functionalized polymers^35^,^36^. It is worth noting that the speciation diagram (Fig. 7) also indicates that desorption of manganese from amidoxime-based sorbents could be difficult if eluents of high pH are used because the manganese complexes with glutarimidedioxime are most stable at higher pH. This would not facilitate the reusability of the sorbents. The sorption of uranium by amidoxime-based sorbents may not be affected because the stability constants for the complexes of uranium and glutarimidedioxime are at least six orders of magnitude higher than that for the manganese complex with glutarimidioxime. From this view, we should develop a new sorbents that can graft more glutarimidedioxime molecular to enhance extraction of uranium.

In summary, complexation of manganese with glutarimidedioxime in aqueous solutions is both enthalpy and entropy driven. The binding strength of glutarimidedioxime with Mn(II) and other cations correlates very well with the charge density of the cations, implying that the bonding is predominantly ionic in nature. The interpretation of two glutarimidedioxime ligands coordinate to manganese can assist redox of Mn(II/III). At seawater pH (8.3), about ~15% of equilibrium manganese complex with glutarimidedioxime, indicating that manganese could compete to some degree with uranium for sorption sites on amidoxime-base sorbents.

## Methods

### Chemicals

All chemicals were reagent-grade or higher. Milli-Q water was used in preparations of all solutions. Glutarimidedioxime (H_2_A, where A^2−^ stands for the fully deprotonated ligand anion) was synthesized according to previous procedures^17^. A stock solution of H_2_A was prepared by dissolving the desired amount of H_2_A in the solution of NaCl (≥98%, Aladdin). Working solutions of NaOH and HCl in NaCl were standardized by titration with potassium hydrogen phthalate (99.95–100.05%, Alfa Aesar) and Trizma base (Crystalline, Sigma), respectly. Carbonate contamination in NaOH titrant solution is less than 0.5% after blank acid-base titrations. Solutions of Mn^2+^ were prepared by dissolving MnCl_2_ (99.99%, Aldrich) in hydrochloric acid. The concentrations of Mn^2+^ and hydrochloric acid in the stock solutions were determined by atomic absorption spectroscopy (AAalyst800, PerkinElmer, USA) and Gran’s titration, respectively. The ionic strength of all working solutions was maintained at 0.5 mol∙L^−1^ (NaCl).

### Potentiometry

The potentiometric vessel consists of a 100 mL glass cell with a lid. Both the cell and the lid are water-jacketed so that the cell temperature can be maintained at 25 °C by water circulating from a constant temperature bath. Experiments are protected by argon throughout the titration to avoid the contamination of carbon dioxide. Argon was passed through a series of solutions including 1.0 mol∙L^−1^ NaOH and 0.5 mol∙L^−1^ NaCl solutions before entering the titration cup. Electromotive force (EMF, in millivolts) was measured by a potentiometric titrator (888 Titrando, Metrohm) equipped with a combination pH electrode (6.0259.100 Unitrode, Metrohm). All the EMF data were corrected for a small contribution from the contact junction potential of hydrogen or hydroxide ion. Corrections for the contact junction potential of the glass electrode in the acidic and basic regions can be expressed by Eqs (3) and (4).


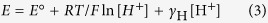



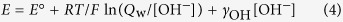


where *R* is the gas constant, *F* is the Faraday constant, and *T* is the temperature in K. *Q*_w_ = [H^+^][OH^−^]. The last terms are the electrode junction potentials (*∆E*_j,H+_ or *∆E*_j,OH−_) for the hydrogen ion (Eq. 3) or the hydroxide ion (Eq. 4), assumed to be proportional to the concentration of the hydrogen or hydroxide ions. The electrode was calibrated before each titration by an acid/base titration with standard HCl and NaOH solution to obtain the electrode parameters of *E*°, 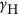, and 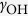. These parameters allow the calculation of hydrogen ion concentrations from the electrode potential in the subsequent titration. Multiple potentiometric titrations were conducted with solution of different concentration of Mn(II) (*C*_Mn_ as total Mn^2+^), ligand (*C*_A_ for the total ligand concentration, including H_2_A, HA^−^ and A^2−^) and acidity (*C*_H_ for total hydrogen ion, where –*C*_H_=*C*_OH_). Usually, about 50 points were collected for each titration. The potentiometric titration data were analyzed to obtain the stability constants of the complexes between the Mn(II) ions and glutarimidedioxime by non-linear regression program.

### Microcalorimetry

The TAM III system consists of a nanocalorimeter, a removable titration ampoule (1.0 mL) with stirring facilities, and a precision syringe pump for titrant delivery. The performance of the calorimeter has been tested by measuring the enthalpy of protonation of tris(hydroxymethyl)-aminomethane. Before each experiment, a dynamic calibration was performed. Multiple titrations with different concentrations of Mn(II), ligand and acidity were performed to reduce the uncertainty of the results. For the complexation of Mn(II) with ligand, 750 μL solution containing Mn(II), the ligand and H^+^ was titrated with a solution NaOH. For each titration, *n* additions were made (5 μL each addition, usually *n* = 50), resulting in *n* experimental values of the heat generated in the reaction cell (*Q*_ex,*j*_, where *j* = 1 to *n*). These values were corrected for the heat of dilution of the titrant (*Q*_dil,*j*_), which was determined in separate runs. The net reaction heat at the *j*-th point (*Q*_r,*j*_) was obtained from the difference: *Q*_r,*j*_ = *Q*_ex,*j*_ − *Q*_dil,*j*_. The observed reaction heat (“partial” or stepwise *Q*) is a function of a number of parameters, including the concentrations of reactants (*C*_H_, *C*_Mn_, and *C*_A_), the equilibrium constants (log*β*) and the enthalpy (∆*H*) of the reactions that occurred in the titration. These data, in conjunction with the protonation constant of H_2_A and the stability constants of Mn(II) complexes obtained by potentiometry, were used to calculate the enthalpy of complexation with Mn(II).

### ESI-mass spectrometry

An LTQ XL Linear Ion Trap Mass Spectrometer (Thermo Fisher) was used to identify the Mn(II) complex with glutaimidedioxime. Water–methanol (1:1 in volume) solutions were used as the spray solvent. Ion dissociation is achieved by multiple energetic collisions with the helium bath gas. In high resolution mode, the instrument has a detection range of 50–2000 *m*/*z* and a resolution of ~0.3 *m*/*z*. Mass spectra were recorded in the positive ion mode.

### Quantum Chemical Calculations

The electronic structure calculations for all of the species were carried out with density functional theory (DFT) methods^37^,^38^ by using Gaussian 09 program. The CAM-B3LYP-D3(BJ) functional^39^ was performed in this study, which included long range correction and London-dispersion correction^40^,^41^,^42^. The Stuttgart effective core potentials (ECPs) and their corresponding valence basis sets were used to describe the manganese atoms^43^. The small-core ECPs represents 10 core electrons in manganese while the remaining 15 electrons were represented by the corresponding valence basis set. The triple split valence basis set 6–311 + G(d) was used to describe hydrogen, carbon nitrogen and oxygen atoms. The default fine grid (75, 302), having 75 radial shells and 302 angular points per shell, was used to evaluate the numerical integration accuracy. All of geometric structures were optimized in aqueous solution while employing the conductor-like polarized continuum model (CPCM)^44^,^45^ model with united atom topological model (UAKS) radii^46^. The harmonic vibrational frequencies were calculated after the geometry optimizations to provide thermodynamic quantities such as the enthalpies (*H*), entropies (*S*) and Gibbs free energies (*G*).

### Electrochemical Measurements

Cyclic voltammetric experiments were carried out on a CHI-600E electrochemical workstation (CH Instruments, Inc. China) driven by CHI software (Version 13.04). A conventional electrochemical three-electrode type cell was used with an Ag/AgCl reference electrode, a platinum wire auxiliary electrode, and a glassy carbon working electrode. Solutions for electrochemical measurement were prepared according the stoichiometric concentrations of the reactants and the stability constants measured by potentiometry in this work.

## Additional Information

**How to cite this article**: Xie, X. *et al*. Complexation of Manganese with Glutarimidedioxime: Implication for Extraction Uranium from Seawater. *Sci. Rep.* **7**, 43503; doi: 10.1038/srep43503 (2017).

**Publisher's note:** Springer Nature remains neutral with regard to jurisdictional claims in published maps and institutional affiliations.

## Supplementary Material

Supplementary Information

## Figures and Tables

**Figure 1:**
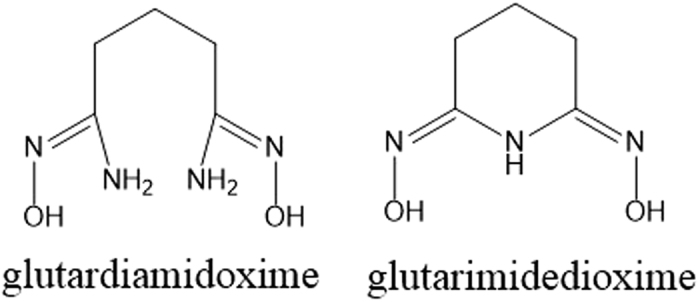
Structures of open chain diamidoxime (left) and cyclic imide dioxime (right).

**Table 1:** Thermodynamic parameters for the complexation of glutarimidedioxime with Mn^2+^ and UO_2_^2+^.

Reaction	log*β*	∆*H* kJ∙mol^−1^	∆*S* J∙mol^−1^∙K^−1^	ref
H^+^ + A^2−^ = HA^−^	12.27 ± 0.03	−36.1 ± 0.5	110 ± 2	17,21
2 H^+^ + A^2−^ = H_2_A	23.15 ± 0.12	−69.7 ± 0.9	202 ± 3	17,21
3 H^+^ + A^2−^ = H_3_A^+^	25.67 ± 0.12	−77.0 ± 6.0	218 ± 14	17,21
Mn^2+^ + H^++^ A^2−^ = MnHA^+^	16.67 ± 0.12	−44.5 ± 0.6	170 ± 4	This work
Mn^2+^ + 2A^2−^ = MnA_2_^2−^	12.78 ± 0.12	−44.6 ± 1.2	95 ± 6	This work
UO_2_^2+^ + H^++^ A^2−^ = UO_2_HA^+^	22.7 ± 1.3	−71.0 ± 6.0	197 ± 14	17
UO_2_^2+^ + 2A^2−^ = UO_2_A_2_^2−^	27.5 ± 2.3	−101 ± 10	188 ± 24	17
