# Supplementary material for: Complexation of Manganese with Glutarimidedioxime: Implication for Extraction Uranium from Seawater
Source: Sci Rep. 2017 Mar 7;7:43503. doi: 10.1038/srep43503 (PMC5339892; doi:10.1038/srep43503)
Supplement: Supplementary Information [file srep43503-s1.doc]

Complexation of Manganese with Glutarimidedioxime: Implication for Extraction Uranium from Seawater

Xiang Xie^1^, Yin Tian^2,*^, Zhen Qin^3^, Qianhong Yu^1^, Hongyuan Wei^1^, Dongqi Wang^4^, Xingliang Li^1,*^ and Xiaolin Wang^1,3,*^

^1^ Institute of Nuclear Physics and Chemistry, China Academy of Engineering Physics, Mianyang, Sichuan 621999, China

^2^ Southwestern Institute of Physics, Chengdu, Sichuan 610041, China

^3^ Institute of Materials, China Academy of Engineering Physics, Mianyang 621900, China

^4^ CAS Key Laboratory of Nuclear Radiation and Nuclear Energy Techniques, and Institute of High Energy Physics, Chinese Academy of Sciences, Beijing, 100049, China.

Address correspondence to

Yin Tian, E-mail: tianyin@swip.ac.cn

Xingliang Li, E-mail: [xingliang@caep.cn](mailto:xingliang@caep.cn)

Xiaolin Wang, E-mail: xlwang@caep.cn


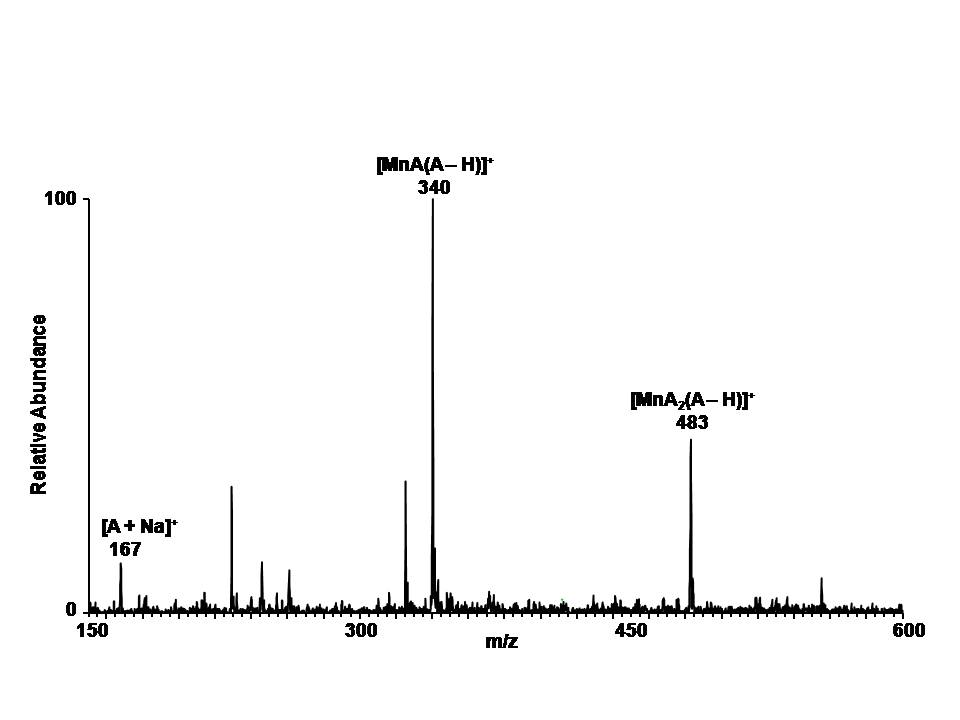


Figure S1 ESI-MS characterization of the Mn(II) complexes with glutaimidedioxime


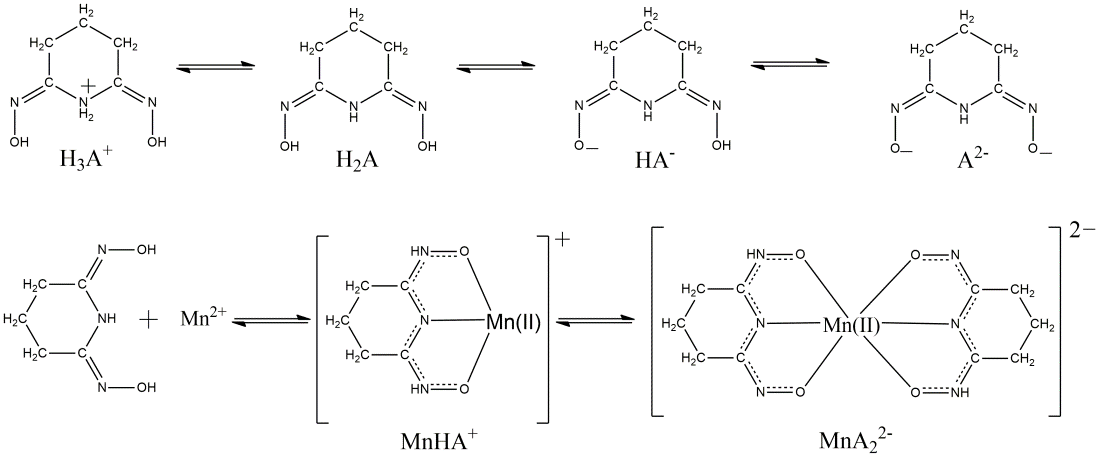


Figure S2 Hypothesized reaction mode for the complexation of Mn(II) with glutaimidedioxime

Table S1 DFT calculations of the thermodynamic parameters for the complexation of glutarimidedioxime with Mn^2+^ in aqueous solution (CPCM, UAKS).

| Reaction | *∆H* / kJ mol^−1^ | *∆S* / J mol^−1^ K^−1^ | *∆G* / kJ mol^−1^ |
| --- | --- | --- | --- |
| Mn^2+^ + HA^−^ = [MnHA]^+^ | −17.49 | 18.58 | −23.04 |
| Mn^2+^ + 2A^2−^ = [MnA_2_]^2−^ | −38.36 | −43.46 | −25.40 |

Table S2 The selected bond lengths (Å) for glutarimidedioxime ligand before and after coordination obtained by the DFT calculations

| species | N_1_‒O_L1_ | C_1_‒N_1_ | N_L_‒C_1_ | C_2_‒N_L_ | N_2_‒C_2_ | O_L2_‒N_2_ |
| --- | --- | --- | --- | --- | --- | --- |
| H_2_A | 1.407 | 1.282 | 1.376 | 1.377 | 1.281 | 1.406 |
| [MnHA(H_2_O)_3_]^+^ | 1.358 | 1.301 | 1.340 | 1.340 | 1.301 | 1.354 |
| [MnA_2_]^2−^ | 1.359 | 1.312 | 1.324 | 1.373 | 1.289 | 1.391 |

Table S3 Mayer bond orders (MBOs) of Mn–Ligand and Mulliken atomic charges (Q) on the Mn and coordinated atom obtained by DFT calculations.*^a^*

| complexes | Mn‒O_L1_ | Mn‒O_L2_ | Mn‒N_L_ | Mn‒O_w_ | Q(Mn) | Q(O_L_) | Q(N_L_) | Q(O_w_) |
| --- | --- | --- | --- | --- | --- | --- | --- | --- |
| [Mn(H_2_O)]^2+^ |  |  |  | 0.120 | 1.467 |  |  | −0.865 |
| [MnHA]^+^ | 0.115 | 0.106 | 0.284 | 0.116 | 1.407 | −0.743 | −0.519 | −0.906 |
| [MnA_2_]^2−^ | 0.086 | 0.138 | 0.144 |  | 1.294 | −0.739 | −0.079 |  |

*^a^* O_L_, N_L_ and O_w_ are coordinated oxygen atom, coordinated oxygen atom in ligand and coordinated oxygen atom in water molecule, respectively.
